# Current Treatment Modalities for Calcified Coronary Artery Disease: A Review Article Comparing Novel Intravascular Lithotripsy and Traditional Rotational Atherectomy

**DOI:** 10.7759/cureus.10922

**Published:** 2020-10-12

**Authors:** Arunima Kaul, Paramvijay Singh Dhalla, Anusha Bapatla, Raheela Khalid, Jian Garcia, Ana S Armenta-Quiroga, Safeera Khan

**Affiliations:** 1 Internal Medicine, California Institute of Behavioral Neurosciences & Psychology, Fairfield, USA; 2 Medicine, California Institute of Behavioral Neurosciences & Psychology, Fairfield, USA; 3 Internal Medicine: Critical Care, California Institute of Behavioral Neurosciences & Psychology, Fairfield, USA

**Keywords:** intravascular lithotripsy, rotational atherectomy, calcified plaque, cad, pad, rotaxus, rotablator, coronary artery intervention

## Abstract

The coronary artery calcium score is considered the most useful marker for predicting coronary events. The high score reflects heavy calcification in the vessel, which is more challenging to treat with the percutaneous intervention (PCI). To prepare this type of heavily calcified lesion intravascular lithotripsy (IVL) technology can be used prior to PCI, which is based on the concept of converting electrical energy into mechanical energy. It harmlessly and selectively disrupts both the shallow and deep deposits of calcium. The balloon-based catheters of this system emit sonic waves that transfer to the adjacent tissue resulting in improvement in vessel compliance with the slightest soft tissue loss. Therefore, making the treatment of calcified lesions more feasible, effective, and also simplify complex lesions. The lesions considered for lithotripsy-enhanced balloon dilation include calcified coronary lesions and peripheral vasculature lesions. This article reviews the use of IVL in calcified coronary artery disease, its advantages, and disadvantages while comparing it with other techniques like high-pressure balloons and rotational atherectomy devices. A thorough search of databases like PubMed and Google Scholar was performed, which uncovered 35 peer review articles. Keywords utilized in the data search were calcified coronary artery disease, coronary lithotripsy, calcification, and calcified atherosclerotic plaque. According to rotational atherectomy and intravascular lithotripsy trials, the latter was safer, mainly by decreasing atheromatous embolization risk. Deciphering these studies, it seems like IVL is better at parameters like procedural and clinical success rate, acute lumen gain, and less residual stenosis except in-hospital major adverse cardiovascular events (MACE), which was better in rotational atherectomy (RA). However, when lesion crossings are present, the atherectomy technique is still considered as first-line therapy. In clinical practice, despite these encouraging data for treating calcified lesions, IVL is grossly underutilized because of substantial costs and perceived significant procedural risk effects on the cardiac rhythm like causing 'shock topics' and asynchronous cardiac pacing. More longer-term clinical data and extensive researches are required to validate its safety and efficiency.

## Introduction and background

Accumulation of calcium salts in body tissues is known as calcification. It usually occurs in bone formation, but calcium can be deposited abnormally in soft tissue, causing it to harden. There are two types of calcifications, one being metastatic calcification, which occurs in normal tissues in which patients are generally hypercalcemic. The other being dystrophic calcification, which occurs secondary to injury or necrosis, while the patients are usually normocalemic. The deposition of calcium in arteries is mainly due to inflammation, vascular injury, and repair. Vascular calcification is the pathological deposition of calcium in vascular structures and is a significant area of study because it commonly affects our aging population and those experiencing diabetes mellitus, dyslipidemia, heart valve disease, and end‐stage renal disease. It is also considered a marker for atherosclerosis and is associated with numerous cardiovascular pathological conditions, like hypertension, heart failure, myocardial ischemia, cardiac hypertrophy, and increased risk of infarction and stroke [1,2].

Vascular calcification is profoundly common in almost all patients with coronary artery disease and, when present, is linked to notable adverse cardiovascular incidents. It is important to differentiate between coronary and peripheral calcifications while considering artery calcification [3]. The osteoblast-like cells act with various contributory factors, like hyperphosphatemia, hypercalcemia, and hyperparathyroidism, often drive medial calcification within the lower extremities peripheral arteries [3]. On a contrary note, the expansion of atherosclerotic coronary calcification has another underlying mechanism, through dysmorphic calcium precipitation induced via chondrocyte-like cells and associated with the appearance of inflammatory factors, such as cytokines. Currently, there is no competent medical therapy that can reverse the calcium deposits in the coronary vessel. The mainstay of treatment is lifestyle changes that can help slow the progression of coronary calcification. These can include smoking cessation, weight loss, alcohol abstinence, along with controlling blood pressure, blood sugar, and lipid levels. Further intervention may be necessary for heavily calcified arteries with severe atherosclerosis that threatens to cause symptoms or disease. This can include procedures like coronary stenting or bypass surgery [4].

Heavily calcified, fibrotic coronary stenosis has traditionally represented a very challenging scenario for percutaneous intervention (PCI), and a common indication for surgical revascularization [5]. This is due to the difficulty of dilating calcified arteries, and it is tough to implant the stents precisely. Very tight calcified lesions may oppose dilation at low balloon inflation pressures or rupture at high pressures. Stent expansion may be subpar due to the high resistance of the calcified plaques causing stent under expansion and malposition. This causes high rates of procedural complications and, consequently, poor clinical outcomes.

Numerous surgical techniques have been used to treat calcified coronary arteries, which includes non-compliant high-pressure balloons, excimer lasers, rotational atherectomy devices, and orbital cutting/scoring balloons [6]. These devices have more significant numbers of procedural complications, such as distal embolization, perforations, and dissections, and depend upon tissue compression and tissue debulking [6]. Furthermore, their success rate decreases with the presence of unconventional, thick, or deep calcifications; besides, induced tissue injury may hasten restenosis and uncontrolled neointimal growth [6-7]. Till now, for improving clinical outcomes, neither specialty balloons nor atherectomy techniques are better than non-compliant high-pressure balloons [6,8,9]. Extracorporeal shock wave lithotripsy is an old technique of using high energy shock waves to treat kidney stones that are now increasingly finding its use in breaking down calcified plaques to assist in stent placement. The intravascular lithotripsy (IVL) system during low-pressure balloon inflation transforms electrical energy into mechanical energy [6,10]. The technology depends upon sonic waves rather than direct vascular tissue injury for plaque modification. While promoting vessel compliance with the least soft tissue impairment, the balloon-based catheter emits sonic waves to the nearby tissue and safely breaks both shallow and deep deposits of calcium [6]. This article reviews extracorporeal shock wave lithotripsy in calcified coronary artery disease, the data available on this therapy so far, and the advantages and disadvantages compared with other techniques like high-pressure balloons and rotational atherectomy devices. It concludes with a discussion about this new technology's future course as its role within cardiac procedures becomes more established.

## Review

Method

Various procedures were followed to ensure a high-quality review of the current literature relevant to the topic. A comprehensive search of databases like PubMed and Google Scholar were performed. Keywords like calcified coronary artery disease, coronary lithotripsy, calcification, and calcified atherosclerotic plaque were used. Only studies pertinent to the main topic, and peer-reviewed articles were used while excluding all non-peer-reviewed articles. Age of the literature included were studies published form the beginning of time till today. Varied study designs were included in this review while excluding any grey literature. The population included were both males and females of ages 18-80 years, excluding children and adolescent populations. All studies included are published in the English language. This research process uncovered 35 peer review articles. 

Discussion

In both asymptomatic and symptomatic cases, coronary artery calcium score is a known tool to predict adverse coronary events. Based on the MESA Study (Multi-Ethnic Study of Atherosclerosis), it was seen that coronary artery calcium is among the most useful markers for predicting atherosclerotic cardiovascular disease (ASCVD) risk. It predicts risk with the same measure of effect in all ethnicities, ages, and both males and females [11]. A primary non-invasive imaging technique used to recognize calcium deposits is coronary CT angiography (CCTA) [4]. While angiography alone underestimates calcium and does not easily allow its quantification, multidetector coronary computed tomography (CT), a non-invasive technique able to measure calcium score and assess prognosis. It is considered by many to be the technique with greatest diagnostic utility [12]. Napkin-ring sign, low CT attenuation, spotty calcification, and the remarkable positive remodeling are the four signs of risky plaques. Acute coronary syndrome and unstable plaques are linked with spotty calcification, which can be detected by CCTA. For increasing PCI's procedural success, CCTA is an essential tool for allowing and planning the procedure by correctly recognizing calcium in coronary lesions and localizing calcium in coronary vessels. The Synergy Between Percutaneous Coronary Intervention with Taxus and Cardiac Surgery (SYNTAX) score is the method utilized to evaluate coronary disease's complexity. The primary factors of high scores are the sites and severity of lesions [13-16].

Interventions for Calcified Coronary Artery Disease

Interventions for calcified coronary artery disease in concert with the progressive aging of the population, the incidence of severe coronary calcification in PCI cases is currently estimated to range between 18% and 26% but is likely to grow. As profoundly calcified coronary lesions are challenging to dilate, it is difficult to deliver and implant stents properly, and they remain a hurdle for percutaneous coronary intervention (PCI). The presence of calcified plaques impairs stent crossing, disrupting drug-polymer from the stent surface, affecting drug delivery and elution, and reducing stent expansion and apposition [17]. This results in increased rates of suboptimal long-term clinical outcomes and periprocedural complications. Though various adjunctive procedural devices have been proposed to increase this particular scenario's success rate, the percutaneous approach to calcified coronary lesions has continuously remained a challenge. Reduced periprocedural complications and increased procedural success are seen with more technologically advanced methods like scoring and cutting balloons, reliable and more efficient non-compliant balloons (NC Balloons), RA, OA, and IVL. However, the current method used for calcified coronary lesions treatment has been rotational atherectomy. Still, novel devices/technologies have entered clinical practice, and combining it with enhanced intravascular imaging, will herald a change in procedural algorithms for the treatment of calcified coronary lesions. NC balloons are the first choice in mild to moderate calcified stenoses due to their high inflation pressures tolerance; they cause balloon expansion in a uniform manner and apply greater forces in a focal segment of a coronary vessel. Whereas, due to exerting intense pressure at the edges, they potentially cause coronary dissections or perforations [18,19]. Cutting balloons consist of microsurgical blades bonded to its surface, which are suitable for making discrete incisions in the atherosclerotic target coronary segment during the balloon's inflation, causing a direct fracture to the luminal calcium. To identify a limited plaque modification, these sorts of methods are easy to employ. However, a subsequent extensive randomized trial revealed that patients treated with conventional balloon angioplasty or with cutting balloon revealed similar acute procedural success in de novo undilatable CAC lesion [9]. Semi-compliant balloons like scoring balloons are encircled by scoring elements, which permits the focal application of the force throughout inflation with a reduction in balloon slippage risks. Though indications of scoring balloons and cutting balloons are similar, however, scoring balloons have a more reliable profile than cutting balloons due to more flexibility, lesser vessel surface injury, and a trivial chance of coronary dissections [20,21]. Atherectomy is a method based on the principles of altering plaque morphology and compliance with plaque-debulking and causing fractures in calcium deposits. Rotational atherectomy is a complex method that utilizes a diamond-tipped burr. It fractures the calcium plaque effectively by its burr's high-speed rotation, which works selectively on calcified tissue and resulting in plaques debulking. Using similar technology to lithotripsy for kidney stones, IVL is a novel technique that disrupts calcified lesions by releasing high-power acoustic shockwaves through a balloon. This method is promising due to its capacity to work on shallow and deep calcium and entrap fractured calcium into the vessel wall, reducing distal embolization. IVL is the single most procedure that may also work on the calcium underneath the stent struts, due to its mechanism of action based on ultrasounds that are not prevented by a stent presence. While all other procedures needed to be applied before stent implantation [22].

The Shockwave Lithotripsy System and Procedure

**Figure 1:**
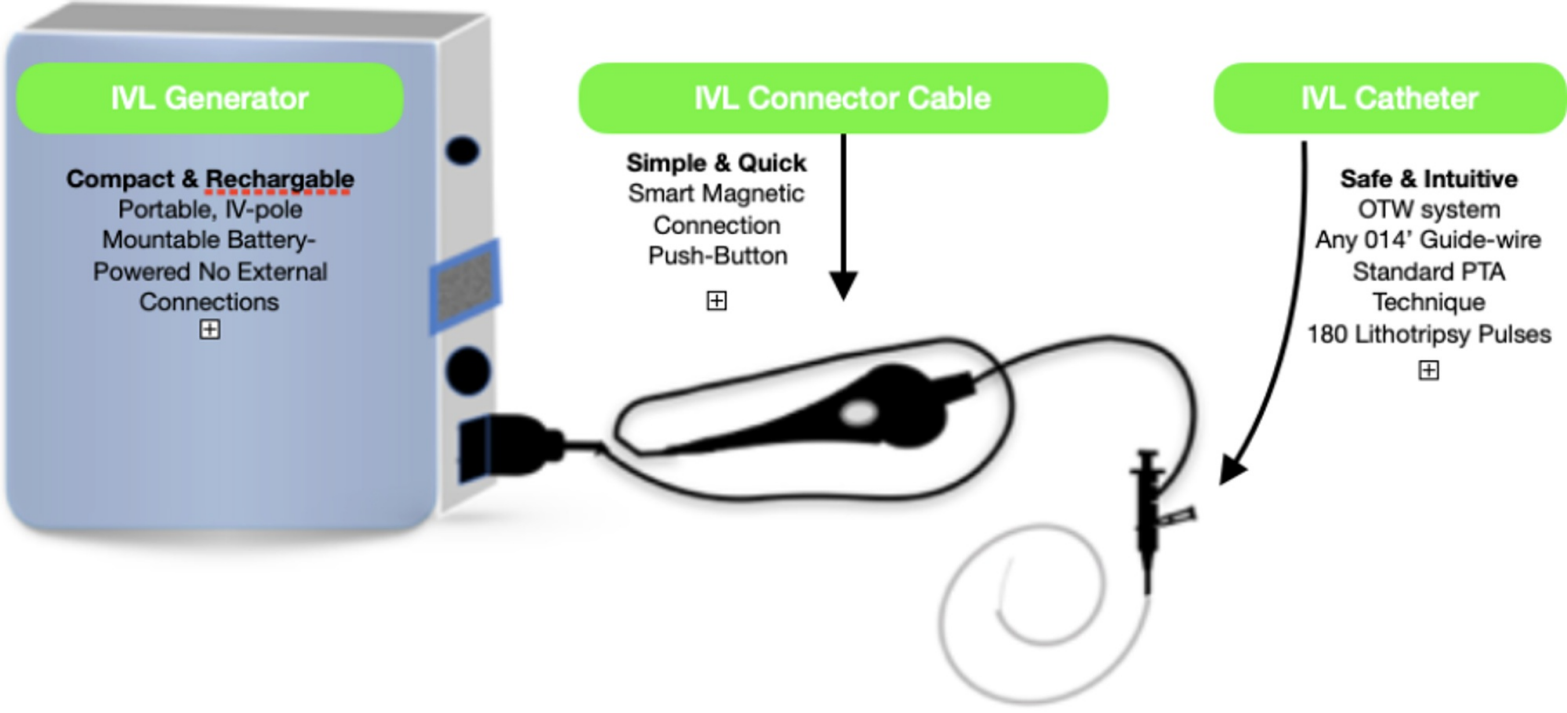
Intravascular lithotripsy equipment

The Coronary IVL System contains a portable, rechargeable generator, a connector cable that comes with a push-button allowing manual, six Fr compatible rapid-exchange controlled delivery of electric pulses, semi-compliant balloon catheter to be utilized following standard angioplasty practice over a 0.014″ guide-wire. In the standard technique, the IVL catheter is placed at the lesion using marker bands angiography. With the integrated balloon inflated at sub nominal pressure at 4 atm by the mixed saline and contrast solution, the fluid between the fully opposed balloon acts as a coupler to facilitate efficient energy transfer of the sonic pressure waves into the vessel wall to reach the calcium. While other treatments can't differentiate between calcium and soft tissue, acoustic pressure waves pass through the soft tissue to impact both intimal and medial calcium. The generator produces 3KW of energy that travels through catheter cables and the connector to the lithotripsy emitters once per second. With emitters along the length of the balloon, a localized field effect is created. A small electrical discharge within the emitters vaporizes the balloon to create a rapidly expanding bubble that generates a sonic pressure wave then collapses within a few microseconds. When the waves impact calcium at nearly 50 atm, they create a series of microfractures. Once a round of ten pulses has been delivered, it is possible to inflate the balloon up to a nominal pressure, which in turn increases balloon compliance and evaluates symmetrical expansion resulting in calcium modification. This calcium modification eventually improves vessel compliance and optimizes stent expansion (Figure 1) [4]. Once lithotripsy has been completed, the operator can proceed with the preferred treatment strategy to optimize outcomes. By making the treatment of calcified lesions more feasible, IVL is simplifying complex procedures.

Indication of the IVL Procedure

The shockwave lithotripsy system is intended to be used for conditions like acute coronary syndromes, unprotected left main calcified stenosis, chronic total occlusions, and stent under-expansion due to underlying calcification. The lesions considered for lithotripsy-enhanced balloon dilation include calcified coronary lesions, whereas in the peripheral vasculature - popliteal, infra-popliteal, the iliac, femoral, iliofemoral, and renal arteries. On the other hand, this technique should not be used if it's not possible to pass 0.014″ guide-wire across the lesion and the procedure is not intended to treat in-stent restenosis or in carotid cerebrovascular arteries. The precautions for this procedure include using only the recommended balloon inflation medium and that the physician should administer appropriate anticoagulant therapy. Risk unique to the device includes - allergy to catheter material, device malfunction or failure, excess heat at the target sites, effects on cardiac rhythm causing 'shock topics,' and asynchronous cardiac pacing have been reported. The adverse effects with standard angioplasty include access site complications, allergy to contrast, bleeding complications, arterial bypass surgery, fracture of guide-wire or device, death, hypertension or hypotension, placement of a stent, infection/sepsis, target vessel stenosis or occlusion, shock/pulmonary edema, renal failure, vascular complications [12].

Rotational Atherectomy

**Figure 2:**
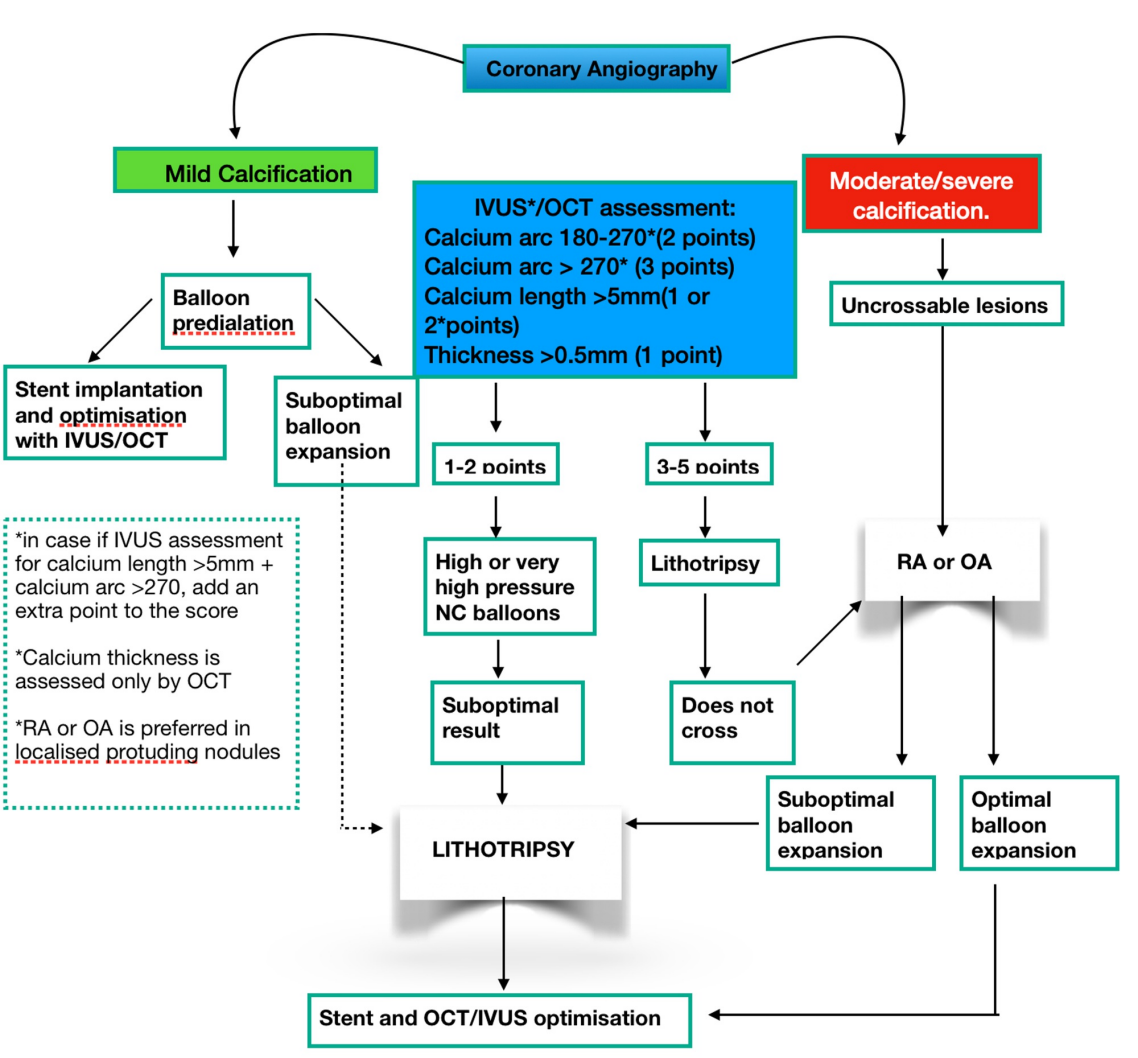
Decisional algorithms for the treatment of calcified coronary lesions IVUS, intravascular ultrasound; OCT, optical coherence tomography; OA, orbital atherectomy; RA, rotational atherectomy

Rotational atherectomy (RA) is an alternative or adjunctive procedure to percutaneous balloon angioplasty; it mechanically ablates resistant or heavily calcified lesions by causing lumen enlargement by physical removal of atherosclerotic plaques and reduction in plaque rigidity, enabling dilation [23]. The three main components of The Rotablator System (Boston Scientific) are as follows: nickel-plated elliptic burr covered with 1.25 to 2.50 mm diameter microscopic diamond crystals; control console, connecting handle, and an advancer that can transfer high rotational speed to the burr which is further connected to a gas-driven turbine [24]. In-stent restenosis decreased markedly throughout the DES era; Consequently, In profoundly calcified stenosis, RA was widely utilized for lesion preparation prior to stent implantation, as confirmed in 2011 by Society for Cardiovascular Angiography and Interventions guidelines (Class C recommendation, Level IIa evidence) [25]. Notable complications of RA are no-reflow or slow flow, which can be treated with intracoronary vasodilators (adenosine, nitroprusside, nicorandil), burr entrapment, transient atrioventricular block, and coronary perforations [26]. Meanwhile, contraindications of RA are dissection, thrombosis, and saphenous vein graft stenosis [27]. The Limitations of rotational atherectomy are the complexity of developing and practicing the system concerning cutting, scoring, or super high-pressure balloons. Moreover, uncommon complications like distal embolization and the no-reflow phenomenon are also evident. The complexity of developing and using the system concerning cutting, scoring, or super high-pressure balloons is the limiting factor of rotational atherectomy. Furthermore, complications like distal embolization and the no-reflow phenomenon are not uncommon. Therefore, to determine the most suitable method according to the circumstances, a decisional algorithm is proposed to guide the interventional cardiologist (Figure 2) [4].

Clinical Outcomes and Safety of IVL- DISRUPT PAD I/ II & PAD III (Peripheral arterial disease)

PAD I/ II first and only core lab adjudicated, long-term study exclusively enrolling heavily calcified lesions. Patients with 85% of severe calcification were included. Results were as follows: Successful delivery of the IVL catheter was achieved in 100% of patients. The post-IVL angiographic acute luminal gain was 2.9 mm, and after drug-eluting stent implantation, residual stenosis was decreased by 24%. Similarly, The Disrupt PAD III Observational Study is a planned, multicenter, nonrandomized, single-arm study designed to evaluate the acute effectiveness and safety of the peripheral IVL system coupled with adjunctive devices in patients who are being treated for calcified lower limb lesions. Enrollment eligibility was if patients had moderate calcification with claudication or chronic limb-threatening ischemia (CLTI, defined as Rutherford category 4-6). Successful delivery of the IVL catheter was achieved in 100% of patients. The acute luminal gain post-IVL angiographic was 3.4 mm, and after drug-eluting stent implantation, residual stenosis was decreased by 23.6%, as demonstrated in (Table 1). The most notable limitation of the Disrupt PAD III being observational study was a single-arm study without a control group as no absolute comparisons can be made to other interventions regarding safety and effectiveness. The trial will track subjects over two years, which will provide data on the longer-term safety and effectiveness of IVL. Hence, only acute procedure results are reported in this study. The PAD III Observational Study is representing the most extensive IVL utilization report in daily clinical practice. Comparing to the prior IVL controlled trials, the use of peripheral IVL in treating severely calcified stenotic lower limb lesions continued to demonstrate consistent acute effectiveness and safety outcomes [28].

**Table 1:** Comparison of intravascular lithotripsy studies with rotational atherectomy study ABI, ankle brachial index; MACE, major adverse cardiovascular events; MI, myocardial infarction; RVD, reference vessel

Multicentre-Single-arm	Disrupt CAD I	Disrupt CAD II	Disrupt PADI/II	Disrupt PAD III (RCT)	ROTAXUS
No. of patients, No. of sites	60 patients, 7 sites	120 patients,15 sites	PAD I: 35 patients, 3 sites PAD II:60 patients, 8 sites	400 patients at 54 sites	240 patients
Inclusion criteria	-De novo moderate/severe calcific coronary lesions -stenosis >50% -RVD 2.5-4.0 mm	-Stabilized acute coronary syndrome -Severe calcification -Diameter stenosis ≥50%, -RVD-2.5-4.0mm -Lesion length ≤32 mm	-Intermittent claudication (Rutherford Class 2-4) -ABI<0.9 -Moderate/severe calcification -SFA/popliteal lesions >70% stenosis -RVD 3.5-7.0 mm -lesion length <150 mm	-Intermittent claudication (Rutherford 2 to 4) -Moderate and severely calcified -Femoropopliteal arteries -RVD 4-7, -Stenosis ≥70%, -Lesion length ≤18 cm occlusive or ≤10 cm CTO	-Stable coronary artery disease -Angina II to IV -Severe calcification -RVD-3.25 -Mean diameter stenosis by visual estimate 83.02 -Lesion length- ≤32 mm
Procedural success	98.3%	100%	100%	100%	92.5%
Clinical success	95%	95.7%	98.9%	-	91.9%
Acute gain	1.7mm	1.6mm	PAD I - 2.9mm PAD II - 3.0mm	3.4-mm	1.56mm
30-day MACE/MAE	5.0%	4.3	PAD I - 0% PADII - 1.7%	-	5%
6-month MACE/MAE	8.3%	-	PAD I - 0% PAD II - 1.1%	-	-
9-month MACE/MAE	-	-	-	-	24.2%

Clinical Outcomes and Safety of Coronary IVL: DISRUPT CAD I & CAD II (Coronary Artery Disease)

Disrupt CAD I was the first prospective multicenter, single-arm trial designed to assess the efficacy and safety of coronary IVL in the treatment of calcified coronary lesions. 60 cases having de novo moderately or severely calcified coronary stenoses in native vessels were enrolled. Device success was 98.3%, and the primary endpoint after stent implantation was residual diameter stenosis <50% without in-hospital MACE (cardiac death/myocardial infarction/target vessel revascularization [TVR]), which was achieved in 95% of cases. According to CAD I, IVL was highly effective, achieving acute luminal gains (1.7±0.6 mm) and residual stenosis (13.3±11.6%) similar to those seen in contemporary drug-eluting stents (DES) studies comprising largely non-calcified lesions. The rate of MACE was 5% and 8% at one and six months, respectively, comprising three non-Q-wave MI and two cardiac deaths deemed unlikely to be related to the index procedure. The absence of vessel perforation, the most fearsome and life-threatening complication of calcific lesions, appears to be a potentially significant advantage of IVL. Still, the absence of large comparative trials limits this anecdotal evidence. The Disrupt CAD II study, a prospective multicenter, the single-arm post-approval study, was conducted at 15 hospitals in nine countries with 120 patients having severe calcified coronary stenoses were enrolled. Successful delivery of the IVL catheter was achieved in 100% of patients. The acute luminal gain post-IVL angiographic was 0.83±0.47 mm, and after drug-eluting stent implantation, residual stenosis was decreased to 7.8±7.1%. Hence, IVL was safely performed with high procedural success and minimal complications. Summing-up, the Disrupt CAD I study demonstrated the usefulness of intravascular lithotripsy (IVL) for the severe coronary artery calcification (CAC) modification. Whereas, CAD II sought to establish the effectiveness and safety of IVL for these lesions [29].

Clinical Outcome and Safety of RA: ROTAXUS

In a recent study in which Rotational Atherectomy was done before Paclitaxel-Eluting Coronary Stent System (TAXUS) Stent Treatment for Complex Native Coronary Artery Disease (ROTAXUS), 240 cases with calcified lesions were randomized in groups of rotational atherectomy prior to stenting or stenting only (paclitaxel stent). This comparison revealed more notable procedural success in the RA group (92.5% versus 83.3%; p=0.03) with a more desirable acute lumen gain, but at nine months, there was marked higher late lumen loss. Although 30-day MACE was 5 %, nine months MACE was 24.2%, which was much higher than expected [30], as shown in Table 1. However, RA is considered to be the gold-standard technique to prepare heavily calcified lesions prior to stent implantation, especially when the balloon device cannot cross lesions. In high-volume centers, it is limited to expert operators, and in Europe, it is employed in only 1%-3% of PCIs [31], apparently due to potential complexities and the expenses, which are not negligible, or when there is weak insurance recruitment. A comparison of IVL studies with RA study is made in Table 1 [12].

While comparing Latest Disrupt CAD II vs. ROTAXUS,120 vs. 240 patients were enrolled, respectively. Successful delivery and use of the IVL catheter were achieved in 100% patients, whereas 92.5% with RA. The post-IVL angiographic acute luminal gain was 1.63±0.49 vs. acute lumen gains 1.56 ± 0.43 in RA, and after drug-eluting stent implantation, residual stenosis with IVL was 7.8±7.1% vs. 10.79 ± 5.61% in RA. The IVL clinical success rate of 95.7% vs. 91.9% in RA. CAD II demonstrated 5.8% in-hospital and 4.3% 30-day MACE vs 4.3% In-hospital and 24.2% 9-month MACE in RA [12,28,30]. The major interpretations from these studies are as follows: Firstly, with the IVL catheter crossing the lesion and delivering therapy in all cases, it was demonstrated that a feasible frontline tool for CAC plaque modification. Secondly, IVL was highly effective. Stenosis reduced severely calcified coronary arteries to a residual of <8% with an acute gain of 1.6 mm and facilitating the delivery of stents in all cases. Thirdly, In-hospital MACE was less in the ROTAXUS trial, but the long-term MACE was remarkably high compared to the IVL studies. Hence IVL was relatively safer with no reported type D to F dissections, perforations, abrupt closure, or slow flow/no-reflow. Fourthly, the IVL mechanism of action was shown to be intraplaque calcium fracture, thereby facilitating stent expansion and modifying vascular compliance [32]. Between the ROTAXUS and CAD I trial, the 30-day MACE was the same, but on comparing their long-term outcome, CAD I trial at six months showed a MACE of 8.3%, While the ROTAXUS at nine months showed a MACE of 24.2%. This points to the fact that IVL may have a lower long-term MACE while comparing it to the RA procedure. 

Benefits of Intravascular lithotripsy over atherectomy are as follows: Firstly, unlike atherectomy, no specific training is required in IVL, as the device is delivered similar to standard catheter-based PCI. Secondly, atheromatous embolization risk may be lower than free debulking devices since IVL therapy is balloon-based. Thirdly, in IVL, energy is distributed uniformly across the lithotripsy emitter. Hence not subject to guide-wire during plaque modification addressing calcium irrespective of its circumferential location. Fourthly, IVL delivers circumferential ultrashort pulses of high-intensity acoustic energy, which results in effective circumferential modification of calcific atheroma by its compressive and decompressive components. Unlike traditional balloon technology, which is reliant on static barometric pressure [33,34]. Fifthly, side-branch protection utilizing a guide-wire may be performed smoothly using IVL, without risk of wire entrapment or severing as may occur with rotational or orbital atherectomy. Lastly, IVL is typically performed at low atmospheric pressure balloon inflation, minimizing mechanical vascular trauma, whereas standard and specialty balloons are inflated at high atmospheric pressure to modify calcium (Table 2) [12].

**Table 2:** Comparison of rotational atherectomy and intravascular lithotripsy in severe coronary calcification

	Rotablator	Intravascular Lithotripsy
Guidewire Size	0.09” Proprietary wire	0.014” Wire of choice
Wire bias	Calcium modification wire-bias dependent	Balloon inflation eliminates wire bias, providing circumferential calcium modification
Lesion crossing	1st line for balloon uncrossable lesions	Higher crossing profile than contemporary balloons
Side branch protection	Side branch wire must be removed during atherectomy	No interaction with side branch wire
Perforation	Accepted risk for atherectomy, higher in tortuous anatomy	No recorded perforations
Distal embolizations	Atherectomy actively liberated atherosclerotic debris	Theoretically same risk as contemporary angioplasty balloon
Plaque ablation	Dependent on selected burr size.	No plaque ablation
Bradyarrhythmias	Temporary pacemaker standard of care in dominant coronary atherectomy	No recorded arrhythmia
Effect of deep calcium	Atherectomy impacts on superficial calcium only	Theoretically modifies deep calcium

Interpreting these studies, it seems like IVUS is better at parameters like procedural and clinical success rate, acute lumen gain, and less residual stenosis except in hospital mace, which was better in RA. However, RA's nine months MACE was too high compared to older IVL studies like CAD I. Thus, pending long-term results from the present DISRUPT CAD II, and additional studies can compare better outcomes. Disrupt CAD I & II demonstrated the safety and effectiveness of IVL to modify these lesions. Hence, IVL as a new therapeutic modality for the management of severe CAC. However, if there is difficulty crossing the lesion with even contemporary low-profile balloon catheters, atherectomy will remain the first-line therapy [17]. Our study has some limitations, like a few studies were done on intravascular coronary lithotripsy, and the studies performed were having a small sample size with no long-term follow-up information. MACE was also not analyzed periodically, which is needed to gives a better idea of the cardiac adverse effects of the procedure. A study showing the Outcomes of IVL with different types of stents has also not been conducted yet. There was no randomized control trial about RA vs. IVL so that we could correlate these findings with real-life scenarios.

## Conclusions

To tackle CAC for revascularization, coronary intravascular lithotripsy is a promising new treatment modality. IVL before stent implantation was performed safely with a low rate of complications and high procedural success then RA. In clinical practice, despite these encouraging data, IVL for treating calcified lesions is grossly underutilized because of substantial costs and perceived greater procedural risk effects on the cardiac rhythm like causing 'shock topics' and asynchronous cardiac pacing. To assess its advantage against other currently available calcium-modifying devices, randomized controlled clinical trials are needed. In partially successful or unsuccessful treatment, devices like RotaTripsy treatment (a sequential combination of Rotational atherectomy and Intravascular lithotripsy) can be employed. Numerous extensive researches with long-term clinical data are necessary to prove this technique's safety and efficacy with careful attention to the impacts on vessel healing response and cardiac conduction, which will help accumulate more evidence for using these game-changing techniques with certainty.
